# Crystal structure, Hirshfeld surface analysis and DFT study of (2*Z*)-2-(2,4-di­chloro­benzyl­idene)-4-[2-(2-oxo-1,3-oxazolidin-3-yl)eth­yl]-3,4-di­hydro-2*H*-1,4-benzo­thia­zin-3-one

**DOI:** 10.1107/S2056989019004250

**Published:** 2019-04-09

**Authors:** Brahim Hni, Nada Kheira Sebbar, Tuncer Hökelek, Lhoussaine El Ghayati, Younes Bouzian, Joel T. Mague, El Mokhtar Essassi

**Affiliations:** a Laboratoire de Chimie Organique Hétérocyclique URAC 21, Pôle de Compétence Pharmacochimie, Av. Ibn Battouta, BP 1014, Faculté des Sciences, Université Mohammed V, Rabat, Morocco; b Laboratoire de Chimie Bioorganique Appliquée, Faculté des sciences, Université Ibn Zohr, Agadir, Morocco; c Department of Physics, Hacettepe University, 06800 Beytepe, Ankara, Turkey; d Department of Chemistry, Tulane University, New Orleans, LA 70118, USA; e Moroccan Foundation for Advanced Science, Innovation and Research (MASCIR), Rabat, Morocco

**Keywords:** crystal structure, di­hydro­benzo­thia­zine, oxazole, π-stacking, Hirshfeld surface

## Abstract

The title compound, C_20_H_16_Cl_2_N_2_O_3_S, is built up from a di­hydro­benzo­thia­zine moiety linked by –CH– and –C_2_H_4_– units to 2,4-di­chloro­phenyl and 2-oxo-1,3-oxazolidine substituents, where the oxazole ring and the heterocyclic portion of the di­hydro­benzo­thia­zine unit adopt envelope and flattened-boat conformations, respectively. The 2-carbon link to the oxazole ring is nearly perpendicular to the mean plane of the di­hydro­benzo­thia­zine unit. In the crystal, the mol­ecules form stacks extending along the normal to (104) with the aromatic rings from neighbouring stacks inter­calating to form an overall layer structure. The Hirshfeld surface analysis of the crystal structure indicates that the most important contributions for the crystal packing are from H⋯H (28.4%), H⋯Cl/Cl⋯H (19.3%), H⋯O/O⋯H (17.0%), H⋯C/C⋯H (14.5%) and C⋯C (8.2%) inter­actions. Weak hydrogen-bonding and van der Waals inter­actions are the dominant inter­actions in the crystal packing. Density functional theory (DFT) optimized structures at the B3LYP/ 6–311 G(d,p) level are compared with the experimentally determined mol­ecular structure in the solid state. The HOMO—LUMO behaviour was elucidated to determine the energy gap.

## Abstract 2

In the title compound, the heterocyclic portion of the di­hydro­benzo­thia­zine unit adopts a flattened-boat conformation, while the oxazolidine ring adopts an envelope conformation. The 2-carbon link to the oxazole ring is perpendicular to the best plane through the di­hydro­benzo­thia­zine unit. In the crystal, the mol­ecules form stacks extending along the normal to (104) through π-stacking inter­actions between the two carbonyl groups and inversion-related oxazole rings. Aromatic rings from neighbouring stacks inter­calate to form an overall layer structure.

## Chemical context   

Compounds containing a 1,4-benzo­thia­zine backbone have been studied extensively both in academic and industrial laboratories. These mol­ecules exhibit a wide range of biological applications indicating that the 1,4-benzo­thia­zine moiety is a template potentially useful in medicinal chemistry research and therapeutic applications such as anti­pyretic (Warren & Knaus, 1987 ▸), anti-microbial (Armenise *et al.*, 2012 ▸; Rathore & Kumar, 2006 ▸; Sabatini *et al.*, 2008 ▸) , anti-viral (Malagu *et al.*, 1998 ▸), herbicide (Takemoto *et al.*, 1994 ▸), anti-cancer (Gupta & Kumar, 1986 ▸) and anti-oxidant (Zia-ur-Rehman *et al.*, 2009 ▸) areas. They have also been reported as precursors for the syntheses of new compounds (Vidal *et al.*, 2006 ▸) possessing anti-diabetic (Tawada *et al.*, 1990 ▸) and anti-corrosion activities (Ellouz *et al.*, 2016*a* ▸,*b* ▸). 1,4-Benzo­thia­zine-containing compounds are important because of their potential applications in the treatment of diabetes complications, by inhibiting aldose reductase (Aotsuka *et al.*, 1994 ▸). They are also used as analgesics (Wammack *et al.*, 2002 ▸) and and antagonists of Ca^2+^ (Fujimura *et al.*, 1996 ▸). As a continuation of our previous work on the syntheses and the biological properties of new 1,4-benzo­thia­zine derivatives (Sebbar *et al.*, 2016*a* ▸,*b* ▸; Ellouz *et al.*, 2015*a* ▸,*b* ▸, 2017*a* ▸,*b* ▸), we report herein on the synthesis and the mol­ecular and crystal structures of the title compound, (I), along with the Hirshfeld surface analysis and the density functional theory (DFT) calculations.
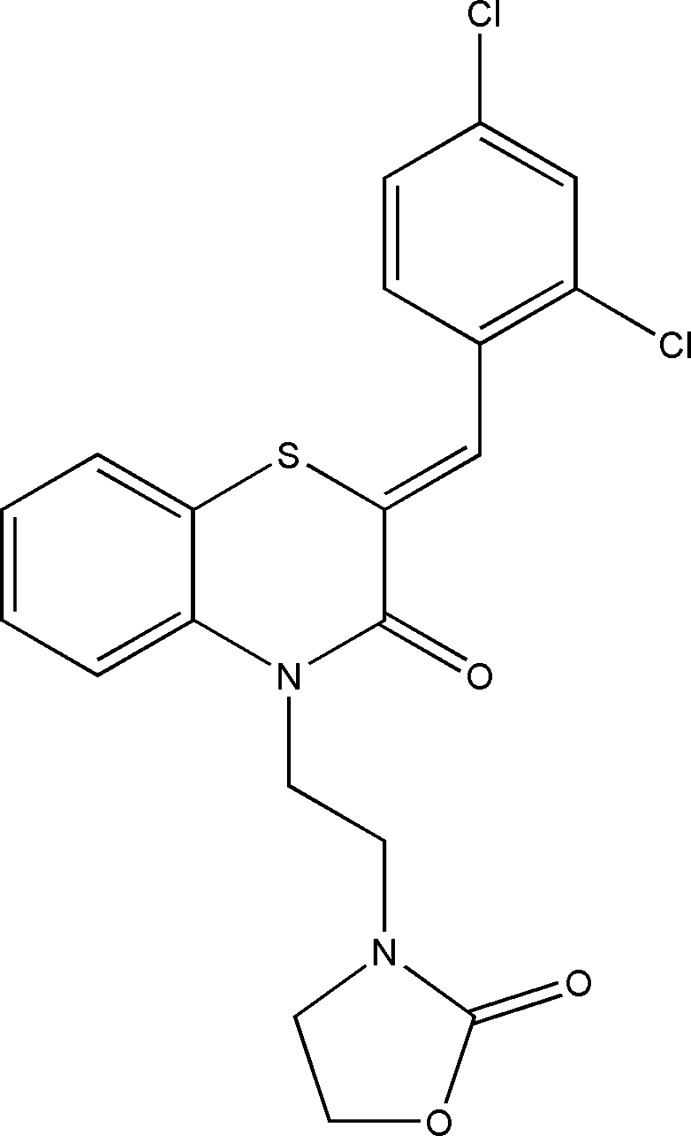


## Structural commentary   

**Figure 1:**
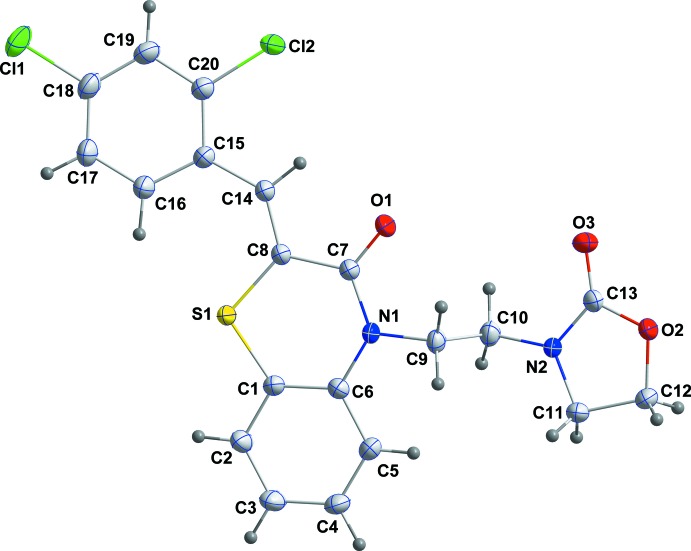
The mol­ecular structure of the title compound with the atom-numbering scheme. Displacement ellipsoids are drawn at the 50% probability level.

The title compound, (I), is built up from a di­hydro­benzo­thia­zine moiety linked by –CH– and C_2_H_2_– units to 2,4-di­chloro­phenyl and 2-oxo-1,3-oxazolidine substituents, respectively (Fig. 1 ▸). The benzene ring, *A* (C1–C6), is oriented at a dihedral angle of 11.27 (6)° with respect to the phenyl ring *D* (C15–C20), ring. A puckering analysis of the heterocyclic portion (ring *B*; S1/N1/C1/C6–C8) of the di­hydro­benzo­thia­zine unit gave the parameters *Q*_T_ = 0.1206 (14) Å, *q*_2_ = 0.1190 (14) Å, *q*_3_ = −0.0174 (16) Å, φ = 178.2 (8)° and θ = 98.4 (8)°, indicating a flattened-boat conformation. A similar analysis for the oxazolidine ring *C* (O2/N2/C11–C13) yielded *q*_2_ = 0.1125 (18) Å and φ_2_ = 45.7 (9)°, indicating an envelope conformation with atom C12 at the flap position and at a distance of 0.175 (2) Å from the best plane of the other four atoms. The C9/C10 chain *C* is essentially perpendicular to the di­hydro­benzo­thia­zine unit, as indicated by the C6—N1—C9—C10 torsion angle of 90.61 (19)°. In the heterocyclic ring *B*, the C1—S1—C8 [104.29 (8)°], S1—C8—C7 [121.39 (12)°], C8—C7—N1 [120.77 (14)°], C7—N1—C6 [126.86 (14)°], C6—C1—S1 [123.97 (13)°] and N1—C6—C1 [121.60 (15)°] bond angles are enlarged compared with the corresponding values in the closely related compounds (2*Z*)-2-(4-chloro­benzyl­idene)-4-[2-(2-oxooxazoliden-3-yl)eth­yl]-3,4-di­hydro-2*H*-1,4-benzo­thia­zin-3-one, (II), (Ellouz *et al.*, 2017*a* ▸) and (2*Z*)-2-[(4-fluoro­benzyl­idene]-4-(prop-2-yn-1-yl)-3,4-di­hydro-2*H*-1,4-benzo­thia­zin-3-one, (III), (Hni *et al.*, 2019 ▸), and they are nearly the same as those in (2*Z*)-4-[2-(2-oxo-1,3-oxazolidin-3-yl)eth­yl]-2(phenyl­methyl­idene)-3,4-di­hydro-2*H*-1,4-benzo­thia­zin-3-one, (IV), (Sebbar *et al.*, 2016*a* ▸), where the heterocyclic portions of the di­hydro­benzo­thia­zine units are planar in (IV) and non-planar in (II) and (III).

## Supra­molecular features   

**Figure 2:**
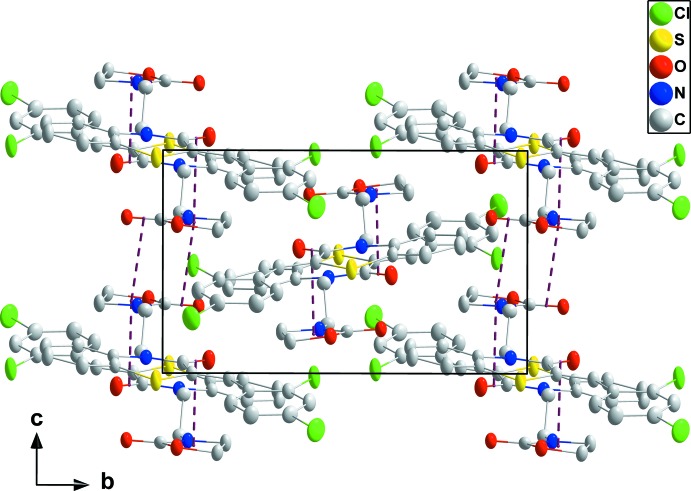
A partial packing diagram viewed along the *a*-axis direction with the π-stacking inter­actions shown by dashed lines.

**Figure 3:**
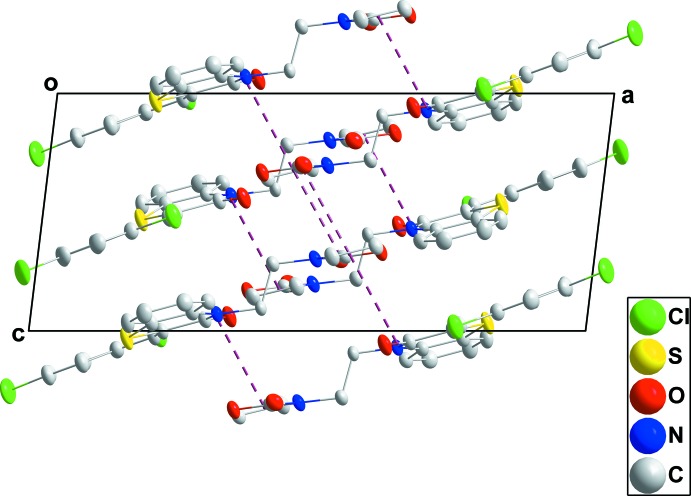
A partial packing diagram viewed along the *b*-axis direction with the π-stacking inter­actions shown by dashed lines.

**Figure 4:**
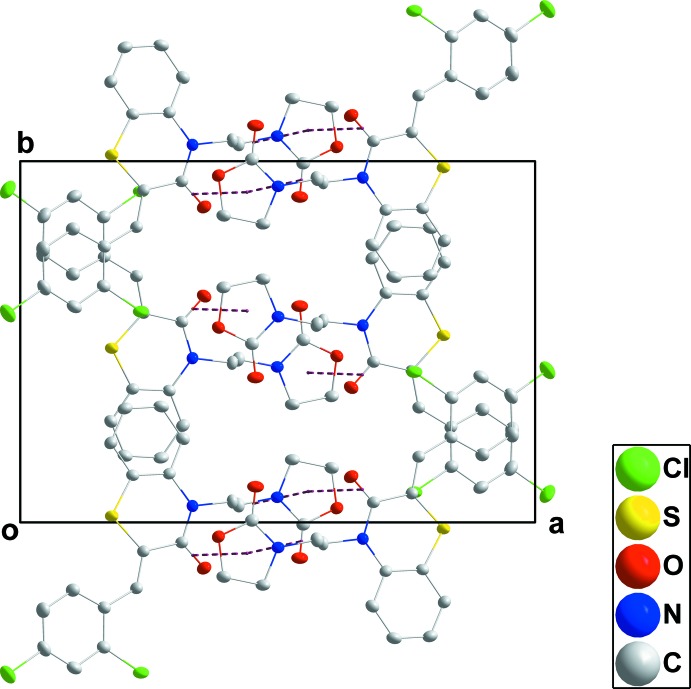
A partial packing diagram viewed along the *c*-axis direction with the π-stacking inter­actions shown by dashed lines.

In the crystal, the mol­ecules form stacks extending along the normal to (104) through π-stacking inter­actions between C7=O1 and the *C* ring at −*x* + 1, −*y* + 1, −*z* + 1 [O1⋯centroid = 3.2744 (16) Å, C7⋯centroid = 3.5448 (18) Å and C7=O1⋯centroid = 92.4 (1)°] and between C13=O3 and the *C* ring at −*x* + 1, −*y* + 1, −*z* [O3⋯centroid = 3.3332 (15) Å, C13⋯centroid = 3.4800 (18) Å and C13=O3⋯centroid = 86.7 (1)°] (Figs. 2 ▸ and 3 ▸). Inter­calation of the aromatic rings between stacks (Fig. 4 ▸) leads to an overall layer structure with the layers approximately parallel to (101) (Fig. 3 ▸).

## Hirshfeld surface analysis   

**Figure 5:**
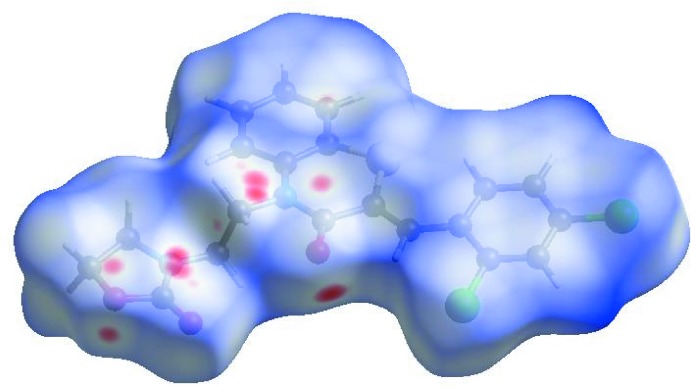
View of the three-dimensional Hirshfeld surface of the title compound plotted over *d*_norm_ in the range −0.1152 to 1.5656 a.u.

**Figure 6:**
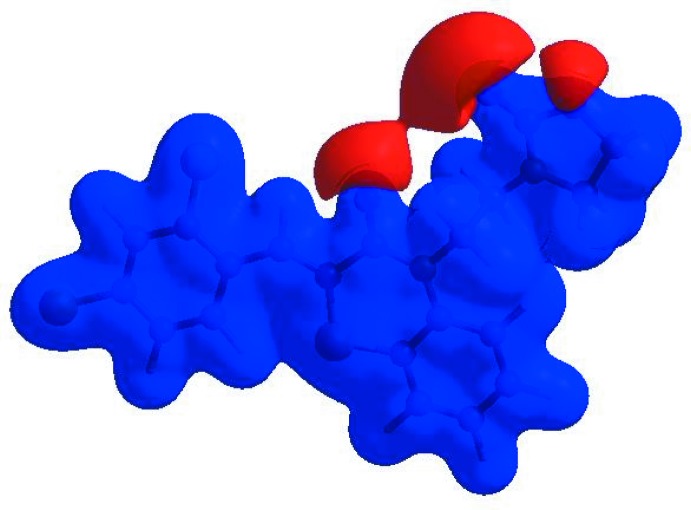
View of the three-dimensional Hirshfeld surface of the title compound plotted over electrostatic potential energy in the range −0.0500 to 0.0500 a.u. using the STO-3 G basis set at the Hartree–Fock level of theory. Hydrogen-bond donors and acceptors are shown as blue and red regions around the atoms, corresponding to positive and negative potentials, respectively.

**Figure 7:**
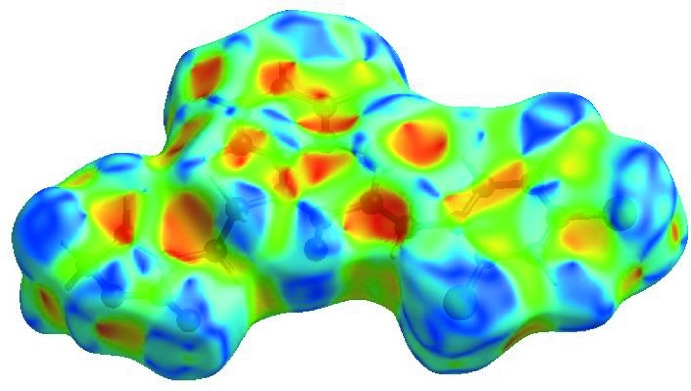
Hirshfeld surface of the title compound plotted over shape-index.

In order to visualize the inter­molecular inter­actions in the crystal of the title compound, a Hirshfeld surface (HS) analysis (Hirshfeld, 1977 ▸; Spackman & Jayatilaka, 2009 ▸) was carried out by using *CrystalExplorer17.5* (Turner *et al.*, 2017 ▸). In the HS plotted over *d*_norm_ (Fig. 5 ▸), the white surface indicates contacts with distances equal to the sum of van der Waals radii, and the red and blue colours indicate distances shorter (in close contact) or longer (distinct contact) than the van der Waals radii, respectively (Venkatesan *et al.*, 2016 ▸). The bright-red spots indicate their roles as the respective donors and/or acceptors; they also appear as blue and red regions corres­ponding to positive and negative potentials on the HS mapped over electrostatic potential (Spackman *et al.*, 2008 ▸; Jayatilaka *et al.*, 2005 ▸), as shown in Fig. 6 ▸. The blue regions indicate positive electrostatic potential (hydrogen-bond donors), while the red regions indicate negative electrostatic potential (hydrogen-bond acceptors). The shape-index of the HS is a tool to visualize the π–π stacking by the presence of adjacent red and blue triangles; if there are no adjacent red and/or blue triangles, then there are no π_ring_–π_ring_ inter­actions. Fig. 7 ▸ clearly suggest that there are no π–π inter­actions in (I).

**Figure 8:**
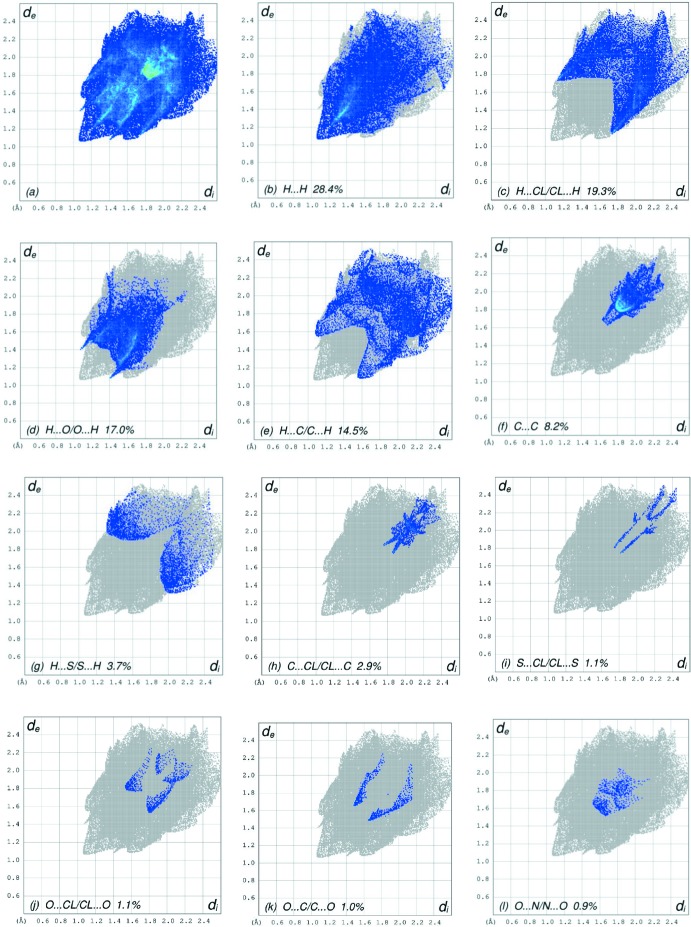
The full two-dimensional fingerprint plots for the title compound, showing (*a*) all inter­actions, and delineated into (*b*) H⋯H, (*c*) H⋯Cl/Cl⋯H, (*d*) H⋯O/O⋯H, (*e*) H⋯C/C⋯H, (*f*) C⋯C, (*g*) H⋯S/S⋯H, (*h*) C⋯Cl/Cl⋯C, (i) S⋯Cl/Cl⋯S, (*j*) O⋯Cl/Cl⋯O, (*k*) O⋯C/C⋯O and (*l*) O⋯N/N⋯O inter­actions. The *d*_i_ and *d*_e_ values are the closest inter­nal and external distances (in Å) from given points on the Hirshfeld surface contacts.

The overall two-dimensional fingerprint plot, Fig. 8 ▸ *a*, and those delineated into H⋯H, H⋯Cl/Cl⋯H, H⋯O/O⋯H, H⋯C/C⋯H, C⋯C, H⋯S/S⋯H, C⋯Cl/Cl⋯C, S⋯Cl/Cl⋯S, O⋯Cl/Cl⋯O, O⋯C/C⋯O and O⋯N/N⋯O contacts (McKinnon *et al.*, 2007 ▸) are illustrated in Fig. 8 ▸ *b*–*l*, respectively, together with their relative contributions to the Hirshfeld surface. The most important inter­action is H⋯H (Table 1 ▸) contributing 28.4% to the overall crystal packing, which is reflected in Fig. 8 ▸ *b* as widely scattered points of high density with the tip at *d*_e_ = *d*_i_ = 1.06 Å. The pair of the scattered points of wings in the fingerprint plot delineated into H⋯Cl/Cl⋯H contacts (19.3% contribution to the HS) have a nearly symmetrical distribution of points, Fig. 8 ▸ *c*, with thin edges at *d*_e_ + *d*_i_ = 2.88 Å. The fingerprint plot delineated into H⋯O/O⋯H contacts (17.0%), Fig. 8 ▸ *d*, has a pair of characteristic wings with a pair of spikes with the tips at *d*_e_ + *d*_i_ = 2.48 Å. In the absence of C—H⋯π inter­actions, the pair of wings in the fingerprint plot delineated into H⋯C/C⋯H contacts (14.5%) have a nearly symmetrical distribution of points, Fig. 8 ▸ *e*, with thick edges at *d*_e_ + *d*_i_ ∼2.66 Å. The C⋯C contacts (8.2%), Fig. 8 ▸ *f*, have an arrow-shaped distribution of points with the tip at *d*_e_ = *d*_i_ ∼1.68 Å. Finally, the H⋯S/S⋯H (Fig. 8 ▸ *g*) and C⋯Cl/Cl⋯C (Fig. 8 ▸ *h*) contacts (3.7% and 2.9%, respectively), and are seen as pairs of wide and thin spikes with the tips at *d*_e_ + *d*_i_ = 3.30 and 3.60 Å, respectively.

**Figure 9:**
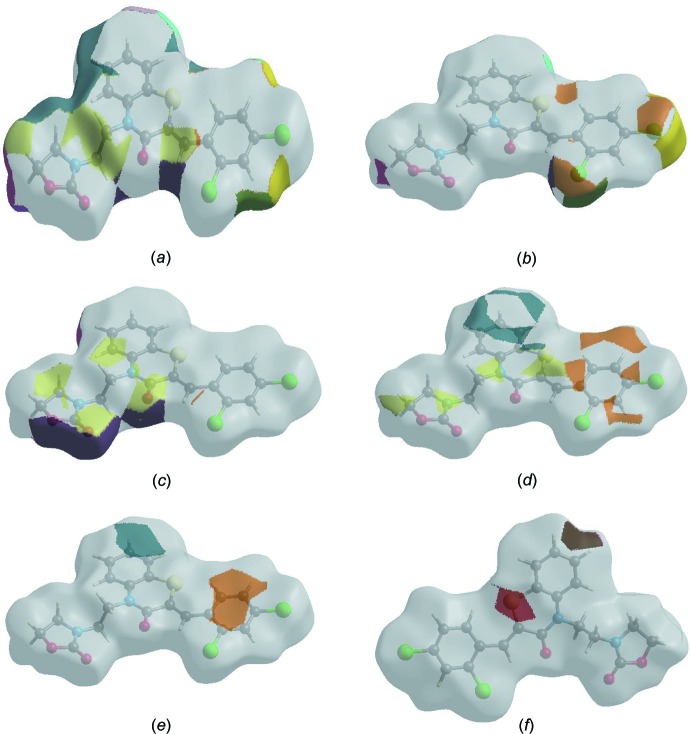
The Hirshfeld surface representations with the function *d*_norm_ plotted onto the surface for (*a*) H⋯H, (*b*) H⋯Cl/Cl⋯H, (*c*) H⋯O/O⋯H, (*d*) H⋯C/C⋯H, (*e*) C⋯C and (*f*) H⋯S/S⋯H inter­actions.

The Hirshfeld surface representations with the function *d*_norm_ plotted onto the surface are shown for the H⋯H, H⋯Cl/Cl⋯H, H⋯O/O⋯H, H⋯C/C⋯H, C⋯C and H⋯S/S⋯H inter­actions in Fig. 9 ▸ *a*–*f*, respectively.

The Hirshfeld surface analysis confirms the importance of H-atom contacts in establishing the packing. The large number of H⋯H, H⋯C/C⋯H, H⋯C/C⋯H and H⋯O/O⋯H inter­actions suggest that van der Waals inter­actions and hydrogen bonding play the major roles in the crystal packing (Hathwar *et al.*, 2015 ▸).

## DFT calculations   

**Figure 10:**
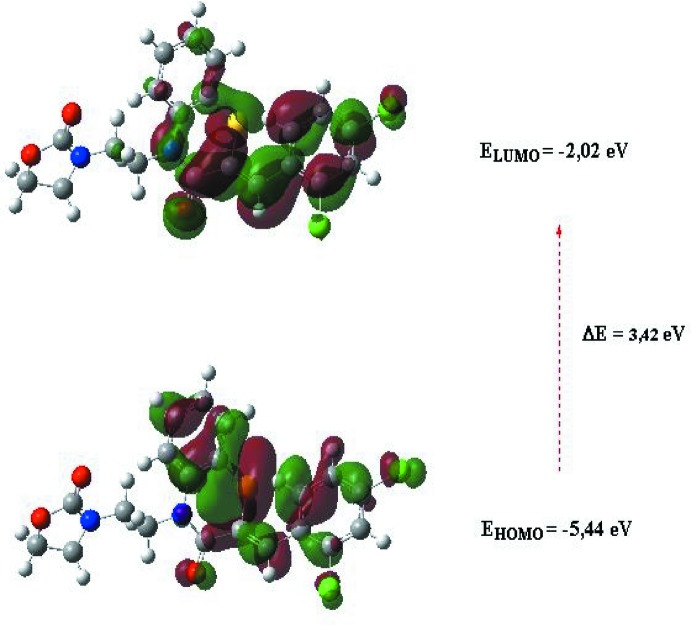
The energy-band gap of the title compound.

The optimized structure of the title compound, (I), in the gas phase was generated theoretically *via* density functional theory (DFT) using standard B3LYP functional and 6–311 G(d,p) basis-set calculations (Becke, 1993 ▸) as implemented in *GAUSSIAN 09* (Frisch *et al.*, 2009 ▸). The theoretical and experimental results were in good agreement. The highest-occupied mol­ecular orbital (HOMO), acting as an electron donor, and the lowest-unoccupied mol­ecular orbital (LUMO), acting as an electron acceptor, are very important parameters for quantum chemistry. When the energy gap is small, the mol­ecule is highly polarizable and has high chemical reactivity. The electron transition from the HOMO to the LUMO energy level is shown in Fig. 10 ▸. The HOMO and LUMO are localized in the plane extending from the whole (2*Z*)-2-[(2,4-di­chloro­phen­yl)methyl­idene]-4-[2-(2-oxo-1,3-oxa­zolidin-3-yl)eth­yl]3,4-di­hydro-2*H*-1,4- benzo­thia­zin-3-one ring. The energy band gap [Δ*E* = *E*_LUMO_ − *E*_HOMO_] of the mol­ecule is about 3.42 eV, and the frontier mol­ecular orbital energies, *E*_HOMO_ and *E*_LUMO_ are −5.44 and −2.02 eV, respectively.

## Database survey   

**Figure 11:**
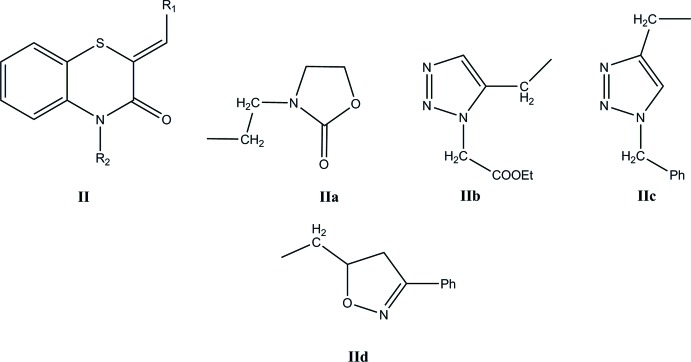
Related structures.

A search of the Cambridge Crystallographic Database (Groom *et al.*, 2016 ▸; updated to Nov. 2018) using the fragment **II** (*R*_1_ = Ph, *R*_2_ = C; Fig. 11 ▸) gave 14 hits with *R*_1_ = Ph and *R*_2_ = CH_2_COOH (Sebbar *et al.*, 2016*c* ▸), *n*-octa­decyl (Sebbar *et al.*, 2017*a* ▸), CH_2_C≡CH (Sebbar *et al.*, 2014*a* ▸), **IIa** (Sebbar *et al.*, 2016*a* ▸), CH_2_COOEt (Zerzouf *et al.*, 2001 ▸), **IIb** (Ellouz *et al.*, 2015*a* ▸), *n*-Bu (Sebbar *et al.*, 2014*b* ▸), **IIc** (Sebbar *et al.*, 2016*d* ▸), Me (Ellouz *et al.*, 2015*b* ▸) and **IId** (Sebbar *et al.*, 2015 ▸). In addition, there are structures with *R*_1_ = 4-ClC_6_H_4_ and *R*_2_ = CH_2_Ph2 (Ellouz *et al.*, 2016*c* ▸), *n*-Bu (Ellouz *et al.*, 2017*a* ▸), **IIa** (Ellouz *et al.*, 2017*c* ▸) and *R*_1_ = 2-ClC_6_H_4_, *R*_2_ = CH_2_C≡CH (Sebbar *et al.*, 2017*b* ▸). In the majority of these, the heterocyclic ring is quite non-planar with the dihedral angle between the plane defined by the benzene ring plus the nitro­gen and sulfur atoms and that defined by nitro­gen and sulfur and the other two carbon atoms separating them ranging from *ca* 29° in CH_2_C≡CH (Sebbar *et al.*, 2014*a* ▸), to 36° in **IId** (Sebbar *et al.*, 2015 ▸), which includes the value of *ca* 30° for 2*H*-1,4-benzo­thia­zin-3(4*H*)-one (WAKLUQ 01; Merola, 2013 ▸). The other three (**IIa**, **IIc** and *R*_1_ = 4-ClC_6_H_4_ and *R*_2_ = CH_2_Ph2; Ellouz *et al.*, 2016*c* ▸) have the benzo­thia­zine unit nearly planar with a corresponding dihedral angle of *ca* 3–4°. In the case of **IIa**, the displacement ellipsoid for the sulfur atom shows a considerable elongation perpendicular to the mean plane of the heterocyclic ring, suggesting disorder, and a greater degree of non-planarity, but for the other two, there is no obvious source for the near planarity.

## Synthesis and crystallization   

Tetra-*n*-butyl­ammonium bromide (0.1 mmol), 2.20 equiv. of bis­(2-chloro­eth­yl)amine hydro­chloride and 2.00 equiv. of potassium carbonate were added to a solution of (*Z*)-2-(2,4-di­chloro­benzyl­idene)-2*H*-1,4-benzo­thia­zin-3(4*H*)-one (1.5 mmol) in DMF (25 ml). The mixture was stirred at 353 K for 6 h. After removal of salts by filtration, the solution was evaporated under reduced pressure and the residue obtained was dissolved in di­chloro­methane. The remaining salts were extracted with distilled water. The residue obtained was chromatographed on a silica gel column (eluent: ethyl acetate/hexa­ne: 3/2). The isolated solid was recrystallized from ethanol solution to afford colourless crystals **[light yellow in CIF?]** (yield: 67%).

## Refinement   

Crystal data, data collection and structure refinement details are summarized in Table 2 ▸. Hydrogen atoms were located in a difference-Fourier map, and freely refined.

## Supplementary Material

Crystal structure: contains datablock(s) I, global. DOI: 10.1107/S2056989019004250/lh5895sup1.cif

Structure factors: contains datablock(s) I. DOI: 10.1107/S2056989019004250/lh5895Isup2.hkl

Supporting information file. DOI: 10.1107/S2056989019004250/lh5895Isup3.cdx

Supporting information file. DOI: 10.1107/S2056989019004250/lh5895Isup4.cml

CCDC reference: 1906476

Additional supporting information:  crystallographic information; 3D view; checkCIF report

## Figures and Tables

**Table 1:** Selected interatomic distances (Å)

Cl1⋯S1^i^	3.5625 (7)	O3⋯H10*B*	2.61 (2)
Cl2⋯C12^ii^	3.470 (2)	O3⋯H5^ii^	2.78 (2)
Cl2⋯C3^iii^	3.557 (2)	O3⋯H11*B* ^ii^	2.80 (2)
Cl2⋯O2^ii^	3.3371 (13)	O3⋯H9*A* ^v^	2.73 (2)
Cl1⋯H3^iv^	3.01 (3)	O3⋯H9*B* ^v^	2.90 (2)
Cl1⋯H16^i^	2.97 (3)	O3⋯H11*A* ^vi^	2.82 (2)
Cl2⋯H14	2.51 (2)	N2⋯O3^vi^	3.165 (2)
Cl2⋯H4^iii^	3.12 (2)	N2⋯C13^vi^	3.190 (2)
Cl2⋯H12*A* ^ii^	3.15 (2)	N2⋯H9*B* ^v^	2.91 (2)
Cl2⋯H3^iii^	2.97 (3)	C5⋯C10	3.422 (3)
S1⋯N1	3.1231 (14)	C7⋯C12^v^	3.580 (3)
S1⋯C16	3.136 (2)	C9⋯C13^v^	3.287 (2)
S1⋯H16	2.45 (2)	C10⋯C13^vi^	3.369 (2)
O1⋯C10	3.187 (2)	C13⋯C13^vi^	3.320 (2)
O1⋯C12^ii^	3.038 (2)	C5⋯H10*A*	2.97 (2)
O1⋯C12^v^	3.304 (3)	C5⋯H9*A*	2.53 (2)
O2⋯C10^vi^	3.255 (2)	C7⋯H10*B*	2.99 (2)
O2⋯C7^v^	3.143 (2)	C8⋯H16	2.99 (2)
O3⋯N2^vi^	3.165 (2)	C9⋯H5	2.52 (2)
O3⋯C11^vi^	3.328 (3)	C9⋯H9*B* ^v^	2.92 (2)
O3⋯C11^ii^	3.375 (2)	C10⋯H5	2.92 (2)
O3⋯C9^v^	3.196 (2)	C13⋯H9*B* ^v^	2.70 (2)
O1⋯H12*B* ^ii^	2.75 (2)	C14⋯H12*B* ^v^	2.98 (2)
O1⋯H9*B*	2.23 (2)	H5⋯H9*A*	2.06 (3)
O1⋯H10*B*	2.73 (2)	H5⋯H10*A*	2.49 (3)
O1⋯H12*A* ^ii^	2.74 (2)	H9*A*⋯H11*B*	2.58 (3)
O1⋯H12*B* ^v^	2.79 (2)	H9*B*⋯H9*B* ^v^	2.26 (3)
O1⋯H14	2.23 (2)	H10*A*⋯H11*A*	2.58 (3)
O2⋯H10*B* ^vi^	2.75 (2)	H10*B*⋯H12*A* ^vi^	2.45 (3)
O2⋯H4^ii^	2.62 (2)	H12*A*⋯H10*B* ^vi^	2.45 (3)

Symmetry codes: (i) 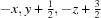; (ii) 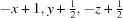; (iii) 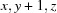; (iv) 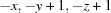; (v) 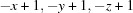; (vi) 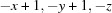.

**Table 2:** Experimental details

Crystal data	
Chemical formula	C_20_H_16_Cl_2_N_2_O_3_S
*M*_r_	435.31
Crystal system, space group	Monoclinic, *P*2_1_/*c*
Temperature (K)	150
*a*, *b*, *c* (Å)	18.4615 (8), 12.8567 (5), 7.9251 (4)
β (°)	96.926 (2)
*V* (Å^3^)	1867.33 (14)
*Z*	4
Radiation type	Cu *K*α
μ (mm^−1^)	4.40
Crystal size (mm)	0.21 × 0.12 × 0.05

Data collection	
Diffractometer	Bruker D8 VENTURE PHOTON 100 CMOS
Absorption correction	Numerical (*SADABS*; Krause *et al.*, 2015 ▸)
*T*_min_, *T*_max_	0.51, 0.80
No. of measured, independent and observed [*I* > 2σ(*I*)] reflections	14033, 3678, 3252
*R*_int_	0.032
(sin θ/λ)_max_ (Å^−1^)	0.619

Refinement	
*R*[*F* ^2^ > 2σ(*F* ^2^)], *wR*(*F* ^2^), *S*	0.035, 0.093, 1.05
No. of reflections	3678
No. of parameters	317
H-atom treatment	All H-atom parameters refined
Δρ_max_, Δρ_min_ (e Å^−3^)	0.37, −0.38

Computer programs: *APEX3* and *SAINT* (Bruker, 2016 ▸), *SHELXT* (Sheldrick, 2015*a* ▸), *SHELXL2018/1* (Sheldrick, 2015*b* ▸), *DIAMOND* (Brandenburg & Putz, 2012 ▸) and *SHELXTL* (Sheldrick, 2008 ▸).

## supplementary crystallographic information

### Crystal data

**Table:**

C_20_H_16_Cl_2_N_2_O_3_S	*F*(000) = 896
*M**_r_* = 435.31	*D*_x_ = 1.548 Mg m^−^^3^
Monoclinic, *P*2_1_/*c*	Cu *K*α radiation, λ = 1.54178 Å
*a* = 18.4615 (8) Å	Cell parameters from 9952 reflections
*b* = 12.8567 (5) Å	θ = 3.5–72.5°
*c* = 7.9251 (4) Å	µ = 4.39 mm^−^^1^
β = 96.926 (2)°	*T* = 150 K
*V* = 1867.33 (14) Å^3^	Column, light yellow
*Z* = 4	0.21 × 0.12 × 0.05 mm

### Data collection

**Table:**

Bruker D8 VENTURE PHOTON 100 CMOS diffractometer	3678 independent reflections
Radiation source: INCOATEC IµS micro–focus source	3252 reflections with *I* > 2σ(*I*)
Mirror monochromator	*R*_int_ = 0.032
Detector resolution: 10.4167 pixels mm^-1^	θ_max_ = 72.5°, θ_min_ = 2.4°
ω scans	*h* = −22→21
Absorption correction: numerical (*SADABS*; Krause *et al.*, 2015)	*k* = −14→15
*T*_min_ = 0.51, *T*_max_ = 0.80	*l* = −9→9
14033 measured reflections	

### Refinement

**Table:**

Refinement on *F*^2^	Primary atom site location: structure-invariant direct methods
Least-squares matrix: full	Secondary atom site location: difference Fourier map
*R*[*F*^2^ > 2σ(*F*^2^)] = 0.035	Hydrogen site location: difference Fourier map
*wR*(*F*^2^) = 0.093	All H-atom parameters refined
*S* = 1.05	*w* = 1/[σ^2^(*F*_o_^2^) + (0.0467*P*)^2^ + 0.9972*P*] where *P* = (*F*_o_^2^ + 2*F*_c_^2^)/3
3678 reflections	(Δ/σ)_max_ = 0.001
317 parameters	Δρ_max_ = 0.37 e Å^−^^3^
0 restraints	Δρ_min_ = −0.38 e Å^−^^3^

### Special details

**Table:**

Geometry. All esds (except the esd in the dihedral angle between two l.s. planes) are estimated using the full covariance matrix. The cell esds are taken into account individually in the estimation of esds in distances, angles and torsion angles; correlations between esds in cell parameters are only used when they are defined by crystal symmetry. An approximate (isotropic) treatment of cell esds is used for estimating esds involving l.s. planes.
Refinement. Refinement of F^2^ against ALL reflections. The weighted R-factor wR and goodness of fit S are based on F^2^, conventional R-factors R are based on F, with F set to zero for negative F^2^. The threshold expression of F^2^ > 2sigma(F^2^) is used only for calculating R-factors(gt) etc. and is not relevant to the choice of reflections for refinement. R-factors based on F^2^ are statistically about twice as large as those based on F, and R- factors based on ALL data will be even larger.

### Fractional atomic coordinates and isotropic or equivalent isotropic displacement parameters (Å^2^)

**Table:**

	*x*	*y*	*z*	*U*_iso_*/*U*_eq_	
Cl1	−0.02396 (3)	0.92135 (4)	0.75404 (8)	0.05021 (17)	
Cl2	0.23463 (3)	0.91630 (3)	0.51698 (8)	0.04067 (15)	
S1	0.17782 (2)	0.48226 (3)	0.53284 (7)	0.03232 (14)	
O1	0.35726 (7)	0.62658 (10)	0.44041 (18)	0.0330 (3)	
O2	0.61496 (6)	0.46053 (10)	0.16700 (16)	0.0267 (3)	
O3	0.54282 (7)	0.59880 (10)	0.19909 (16)	0.0299 (3)	
N1	0.33467 (7)	0.45458 (11)	0.42952 (18)	0.0227 (3)	
N2	0.49920 (8)	0.43048 (11)	0.19710 (19)	0.0240 (3)	
C1	0.21852 (9)	0.36941 (13)	0.4668 (2)	0.0237 (3)	
C2	0.17612 (10)	0.27967 (14)	0.4671 (3)	0.0298 (4)	
H2	0.1287 (13)	0.2873 (18)	0.496 (3)	0.036 (6)*	
C3	0.20375 (10)	0.18467 (15)	0.4260 (3)	0.0328 (4)	
H3	0.1739 (14)	0.125 (2)	0.424 (3)	0.043 (6)*	
C4	0.27384 (10)	0.17928 (14)	0.3800 (2)	0.0299 (4)	
H4	0.2935 (12)	0.1146 (19)	0.352 (3)	0.037 (6)*	
C5	0.31603 (10)	0.26806 (14)	0.3770 (2)	0.0255 (4)	
H5	0.3619 (13)	0.2616 (18)	0.342 (3)	0.035 (6)*	
C6	0.28970 (9)	0.36498 (13)	0.4241 (2)	0.0221 (3)	
C7	0.31385 (9)	0.55557 (13)	0.4532 (2)	0.0238 (3)	
C8	0.23937 (9)	0.57999 (13)	0.5014 (2)	0.0233 (3)	
C9	0.41197 (9)	0.44207 (14)	0.4043 (2)	0.0235 (3)	
H9A	0.4306 (11)	0.3741 (17)	0.457 (3)	0.029 (5)*	
H9B	0.4388 (11)	0.4975 (17)	0.468 (3)	0.028 (5)*	
C10	0.42367 (9)	0.45187 (15)	0.2187 (2)	0.0264 (4)	
H10A	0.3897 (12)	0.4031 (17)	0.146 (3)	0.031 (5)*	
H10B	0.4108 (12)	0.5235 (18)	0.181 (3)	0.033 (6)*	
C11	0.52850 (10)	0.32643 (14)	0.1876 (3)	0.0285 (4)	
H11A	0.4991 (13)	0.2859 (18)	0.099 (3)	0.038 (6)*	
H11B	0.5292 (13)	0.2906 (19)	0.295 (3)	0.043 (6)*	
C12	0.60490 (10)	0.34916 (14)	0.1433 (2)	0.0272 (4)	
H12A	0.6082 (12)	0.3331 (18)	0.024 (3)	0.037 (6)*	
H12B	0.6420 (13)	0.3167 (19)	0.219 (3)	0.039 (6)*	
C13	0.54980 (9)	0.50543 (13)	0.1887 (2)	0.0227 (3)	
C14	0.22602 (9)	0.68205 (14)	0.5250 (2)	0.0246 (4)	
H14	0.2654 (11)	0.7266 (17)	0.504 (3)	0.027 (5)*	
C15	0.16331 (9)	0.73590 (13)	0.5785 (2)	0.0241 (4)	
C16	0.10234 (10)	0.68752 (15)	0.6352 (3)	0.0311 (4)	
H16	0.0973 (13)	0.612 (2)	0.640 (3)	0.044 (7)*	
C17	0.04455 (10)	0.74288 (16)	0.6871 (3)	0.0344 (4)	
H17	0.0037 (15)	0.710 (2)	0.725 (3)	0.050 (7)*	
C18	0.04662 (10)	0.84994 (16)	0.6841 (3)	0.0330 (4)	
C19	0.10510 (10)	0.90291 (15)	0.6301 (3)	0.0325 (4)	
H19	0.1073 (11)	0.9771 (19)	0.626 (3)	0.032 (6)*	
C20	0.16222 (10)	0.84560 (14)	0.5794 (2)	0.0271 (4)	

### Atomic displacement parameters (Å^2^)

**Table:**

	*U*^11^	*U*^22^	*U*^33^	*U*^12^	*U*^13^	*U*^23^
Cl1	0.0335 (3)	0.0432 (3)	0.0774 (4)	0.0150 (2)	0.0207 (3)	−0.0079 (3)
Cl2	0.0354 (3)	0.0187 (2)	0.0718 (4)	−0.00040 (17)	0.0224 (2)	0.0036 (2)
S1	0.0250 (2)	0.0169 (2)	0.0593 (3)	−0.00047 (16)	0.0225 (2)	−0.00057 (19)
O1	0.0279 (6)	0.0224 (7)	0.0522 (8)	−0.0045 (5)	0.0198 (6)	−0.0021 (6)
O2	0.0227 (6)	0.0208 (6)	0.0390 (7)	−0.0020 (5)	0.0131 (5)	−0.0006 (5)
O3	0.0380 (7)	0.0183 (6)	0.0349 (7)	0.0008 (5)	0.0106 (5)	0.0001 (5)
N1	0.0187 (7)	0.0201 (7)	0.0308 (7)	0.0006 (5)	0.0097 (5)	−0.0007 (6)
N2	0.0224 (7)	0.0182 (7)	0.0338 (8)	−0.0002 (5)	0.0131 (6)	−0.0008 (6)
C1	0.0231 (8)	0.0175 (8)	0.0316 (9)	0.0011 (6)	0.0080 (7)	0.0013 (6)
C2	0.0232 (8)	0.0219 (9)	0.0454 (11)	−0.0026 (7)	0.0097 (7)	0.0003 (8)
C3	0.0306 (9)	0.0192 (9)	0.0493 (12)	−0.0039 (7)	0.0079 (8)	−0.0017 (8)
C4	0.0323 (9)	0.0187 (9)	0.0395 (10)	0.0027 (7)	0.0070 (8)	−0.0039 (7)
C5	0.0252 (8)	0.0221 (9)	0.0304 (9)	0.0036 (7)	0.0083 (7)	−0.0007 (7)
C6	0.0220 (8)	0.0183 (8)	0.0268 (8)	−0.0015 (6)	0.0060 (6)	0.0008 (6)
C7	0.0225 (8)	0.0186 (8)	0.0320 (9)	−0.0007 (6)	0.0105 (7)	0.0000 (7)
C8	0.0212 (8)	0.0187 (8)	0.0319 (9)	−0.0005 (6)	0.0103 (6)	0.0012 (6)
C9	0.0167 (7)	0.0272 (9)	0.0274 (8)	0.0014 (7)	0.0063 (6)	−0.0011 (7)
C10	0.0219 (8)	0.0298 (10)	0.0287 (9)	0.0022 (7)	0.0079 (7)	0.0010 (7)
C11	0.0294 (9)	0.0171 (8)	0.0412 (10)	−0.0003 (7)	0.0137 (8)	0.0007 (7)
C12	0.0280 (9)	0.0189 (8)	0.0365 (10)	0.0015 (7)	0.0116 (8)	−0.0030 (7)
C13	0.0257 (8)	0.0212 (8)	0.0224 (8)	−0.0008 (7)	0.0085 (6)	0.0008 (6)
C14	0.0217 (8)	0.0193 (8)	0.0346 (9)	−0.0008 (7)	0.0109 (7)	0.0007 (7)
C15	0.0229 (8)	0.0206 (8)	0.0299 (9)	0.0021 (6)	0.0075 (7)	0.0005 (7)
C16	0.0265 (9)	0.0236 (9)	0.0456 (11)	−0.0001 (7)	0.0143 (8)	−0.0021 (8)
C17	0.0255 (9)	0.0330 (10)	0.0475 (12)	0.0011 (8)	0.0155 (8)	−0.0029 (8)
C18	0.0255 (9)	0.0328 (10)	0.0416 (10)	0.0085 (8)	0.0087 (8)	−0.0040 (8)
C19	0.0311 (10)	0.0226 (9)	0.0445 (11)	0.0062 (7)	0.0070 (8)	−0.0002 (8)
C20	0.0250 (8)	0.0219 (9)	0.0353 (9)	0.0019 (7)	0.0070 (7)	0.0010 (7)

### Geometric parameters (Å, º)

**Table:**

Cl1—C18	1.7385 (19)	C7—C8	1.504 (2)
Cl2—C20	1.7373 (18)	C8—C14	1.352 (2)
S1—C8	1.7321 (17)	C9—C10	1.518 (2)
S1—C1	1.7430 (17)	C9—H9A	1.01 (2)
O1—C7	1.227 (2)	C9—H9B	0.97 (2)
O2—C13	1.364 (2)	C10—H10A	1.01 (2)
O2—C12	1.453 (2)	C10—H10B	0.99 (2)
O3—C13	1.211 (2)	C11—C12	1.522 (2)
N1—C7	1.374 (2)	C11—H11A	0.98 (2)
N1—C6	1.418 (2)	C11—H11B	0.97 (3)
N1—C9	1.473 (2)	C12—H12A	0.97 (2)
N2—C13	1.349 (2)	C12—H12B	0.95 (2)
N2—C11	1.448 (2)	C14—C15	1.455 (2)
N2—C10	1.451 (2)	C14—H14	0.96 (2)
C1—C2	1.394 (2)	C15—C16	1.406 (2)
C1—C6	1.397 (2)	C15—C20	1.411 (2)
C2—C3	1.378 (3)	C16—C17	1.386 (3)
C2—H2	0.94 (2)	C16—H16	0.97 (3)
C3—C4	1.388 (3)	C17—C18	1.377 (3)
C3—H3	0.94 (3)	C17—H17	0.95 (3)
C4—C5	1.384 (3)	C18—C19	1.387 (3)
C4—H4	0.94 (2)	C19—C20	1.385 (3)
C5—C6	1.404 (2)	C19—H19	0.96 (2)
C5—H5	0.93 (2)		
			
Cl1···S1^i^	3.5625 (7)	O3···H10B	2.61 (2)
Cl2···C12^ii^	3.470 (2)	O3···H5^ii^	2.78 (2)
Cl2···C3^iii^	3.557 (2)	O3···H11B^ii^	2.80 (2)
Cl2···O2^ii^	3.3371 (13)	O3···H9A^v^	2.73 (2)
Cl1···H3^iv^	3.01 (3)	O3···H9B^v^	2.90 (2)
Cl1···H16^i^	2.97 (3)	O3···H11A^vi^	2.82 (2)
Cl2···H14	2.51 (2)	N2···O3^vi^	3.165 (2)
Cl2···H4^iii^	3.12 (2)	N2···C13^vi^	3.190 (2)
Cl2···H12A^ii^	3.15 (2)	N2···H9B^v^	2.91 (2)
Cl2···H3^iii^	2.97 (3)	C5···C10	3.422 (3)
S1···N1	3.1231 (14)	C7···C12^v^	3.580 (3)
S1···C16	3.136 (2)	C9···C13^v^	3.287 (2)
S1···H16	2.45 (2)	C10···C13^vi^	3.369 (2)
O1···C10	3.187 (2)	C13···C13^vi^	3.320 (2)
O1···C12^ii^	3.038 (2)	C5···H10A	2.97 (2)
O1···C12^v^	3.304 (3)	C5···H9A	2.53 (2)
O2···C10^vi^	3.255 (2)	C7···H10B	2.99 (2)
O2···C7^v^	3.143 (2)	C8···H16	2.99 (2)
O3···N2^vi^	3.165 (2)	C9···H5	2.52 (2)
O3···C11^vi^	3.328 (3)	C9···H9B^v^	2.92 (2)
O3···C11^ii^	3.375 (2)	C10···H5	2.92 (2)
O3···C9^v^	3.196 (2)	C13···H9B^v^	2.70 (2)
O1···H12B^ii^	2.75 (2)	C14···H12B^v^	2.98 (2)
O1···H9B	2.23 (2)	H5···H9A	2.06 (3)
O1···H10B	2.73 (2)	H5···H10A	2.49 (3)
O1···H12A^ii^	2.74 (2)	H9A···H11B	2.58 (3)
O1···H12B^v^	2.79 (2)	H9B···H9B^v^	2.26 (3)
O1···H14	2.23 (2)	H10A···H11A	2.58 (3)
O2···H10B^vi^	2.75 (2)	H10B···H12A^vi^	2.45 (3)
O2···H4^ii^	2.62 (2)	H12A···H10B^vi^	2.45 (3)
			
C8—S1—C1	104.29 (8)	C9—C10—H10A	110.3 (12)
C13—O2—C12	109.43 (13)	N2—C10—H10B	109.8 (13)
C7—N1—C6	126.86 (14)	C9—C10—H10B	108.4 (13)
C7—N1—C9	114.42 (14)	H10A—C10—H10B	107.1 (17)
C6—N1—C9	118.72 (14)	N2—C11—C12	101.36 (14)
C13—N2—C11	113.07 (14)	N2—C11—H11A	110.5 (13)
C13—N2—C10	123.45 (15)	C12—C11—H11A	112.7 (14)
C11—N2—C10	123.45 (14)	N2—C11—H11B	111.1 (14)
C2—C1—C6	120.75 (16)	C12—C11—H11B	112.3 (14)
C2—C1—S1	115.20 (13)	H11A—C11—H11B	108.8 (19)
C6—C1—S1	123.97 (13)	O2—C12—C11	105.47 (13)
C3—C2—C1	120.61 (17)	O2—C12—H12A	108.2 (14)
C3—C2—H2	122.4 (14)	C11—C12—H12A	110.6 (13)
C1—C2—H2	117.0 (14)	O2—C12—H12B	106.3 (14)
C2—C3—C4	119.33 (17)	C11—C12—H12B	112.9 (14)
C2—C3—H3	119.4 (15)	H12A—C12—H12B	113.0 (19)
C4—C3—H3	121.2 (15)	O3—C13—N2	128.64 (16)
C5—C4—C3	120.53 (17)	O3—C13—O2	122.07 (15)
C5—C4—H4	119.3 (14)	N2—C13—O2	109.30 (14)
C3—C4—H4	120.1 (14)	C8—C14—C15	131.80 (16)
C4—C5—C6	120.93 (16)	C8—C14—H14	113.7 (13)
C4—C5—H5	117.9 (14)	C15—C14—H14	114.5 (13)
C6—C5—H5	121.2 (14)	C16—C15—C20	115.34 (16)
C1—C6—C5	117.79 (15)	C16—C15—C14	125.33 (16)
C1—C6—N1	121.60 (15)	C20—C15—C14	119.31 (16)
C5—C6—N1	120.60 (15)	C17—C16—C15	122.84 (18)
O1—C7—N1	119.71 (15)	C17—C16—H16	114.8 (15)
O1—C7—C8	119.48 (15)	C15—C16—H16	122.4 (15)
N1—C7—C8	120.77 (14)	C18—C17—C16	118.95 (18)
C14—C8—C7	115.16 (15)	C18—C17—H17	118.7 (16)
C14—C8—S1	123.40 (13)	C16—C17—H17	122.4 (16)
C7—C8—S1	121.39 (12)	C17—C18—C19	121.36 (17)
N1—C9—C10	112.06 (14)	C17—C18—Cl1	119.91 (15)
N1—C9—H9A	109.0 (12)	C19—C18—Cl1	118.70 (15)
C10—C9—H9A	113.1 (12)	C20—C19—C18	118.45 (18)
N1—C9—H9B	106.8 (12)	C20—C19—H19	118.9 (13)
C10—C9—H9B	108.7 (12)	C18—C19—H19	122.7 (13)
H9A—C9—H9B	106.9 (16)	C19—C20—C15	123.05 (17)
N2—C10—C9	110.45 (14)	C19—C20—Cl2	116.31 (14)
N2—C10—H10A	110.7 (12)	C15—C20—Cl2	120.64 (13)
			
C8—S1—C1—C2	−175.06 (14)	C11—N2—C10—C9	81.1 (2)
C8—S1—C1—C6	8.14 (18)	N1—C9—C10—N2	−175.21 (14)
C6—C1—C2—C3	0.3 (3)	C13—N2—C11—C12	−8.6 (2)
S1—C1—C2—C3	−176.63 (16)	C10—N2—C11—C12	173.17 (16)
C1—C2—C3—C4	−1.5 (3)	C13—O2—C12—C11	−10.95 (19)
C2—C3—C4—C5	0.6 (3)	N2—C11—C12—O2	11.27 (19)
C3—C4—C5—C6	1.5 (3)	C11—N2—C13—O3	−177.18 (18)
C2—C1—C6—C5	1.8 (3)	C10—N2—C13—O3	1.1 (3)
S1—C1—C6—C5	178.44 (13)	C11—N2—C13—O2	2.2 (2)
C2—C1—C6—N1	−177.45 (16)	C10—N2—C13—O2	−179.56 (15)
S1—C1—C6—N1	−0.8 (2)	C12—O2—C13—O3	−174.76 (16)
C4—C5—C6—C1	−2.7 (3)	C12—O2—C13—N2	5.81 (18)
C4—C5—C6—N1	176.56 (16)	C7—C8—C14—C15	−176.93 (18)
C7—N1—C6—C1	−9.1 (3)	S1—C8—C14—C15	0.3 (3)
C9—N1—C6—C1	172.24 (16)	C8—C14—C15—C16	6.6 (3)
C7—N1—C6—C5	171.68 (17)	C8—C14—C15—C20	−175.08 (19)
C9—N1—C6—C5	−7.0 (2)	C20—C15—C16—C17	0.6 (3)
C6—N1—C7—O1	−173.69 (16)	C14—C15—C16—C17	179.02 (19)
C9—N1—C7—O1	5.0 (2)	C15—C16—C17—C18	−0.3 (3)
C6—N1—C7—C8	8.7 (3)	C16—C17—C18—C19	0.1 (3)
C9—N1—C7—C8	−172.58 (15)	C16—C17—C18—Cl1	−178.22 (16)
O1—C7—C8—C14	0.9 (3)	C17—C18—C19—C20	−0.3 (3)
N1—C7—C8—C14	178.52 (16)	Cl1—C18—C19—C20	178.04 (15)
O1—C7—C8—S1	−176.37 (14)	C18—C19—C20—C15	0.7 (3)
N1—C7—C8—S1	1.2 (2)	C18—C19—C20—Cl2	−178.85 (15)
C1—S1—C8—C14	174.78 (16)	C16—C15—C20—C19	−0.8 (3)
C1—S1—C8—C7	−8.17 (17)	C14—C15—C20—C19	−179.34 (18)
C7—N1—C9—C10	−88.22 (19)	C16—C15—C20—Cl2	178.68 (14)
C6—N1—C9—C10	90.61 (19)	C14—C15—C20—Cl2	0.2 (2)
C13—N2—C10—C9	−97.0 (2)		

Symmetry codes: (i) −*x*, *y*+1/2, −*z*+3/2; (ii) −*x*+1, *y*+1/2, −*z*+1/2; (iii) *x*, *y*+1, *z*; (iv) −*x*, −*y*+1, −*z*+1; (v) −*x*+1, −*y*+1, −*z*+1; (vi) −*x*+1, −*y*+1, −*z*.

## References

[bb1] Aotsuka, T., Hosono, H., Kurihara, T., Nakamura, Y., Matsui, T. & Kobayashi, F. (1994). *Chem. Pharm. Bull.* **42**, 1264–1271. 10.1248/cpb.42.1264 8069975

[bb2] Armenise, D., Muraglia, M., Florio, M. A., Laurentis, N. D., Rosato, A., Carrieri, A., Corbo, F. & Franchini, C. (2012). *Mol. Pharmacol.* **50**, 1178–1188.

[bb3] Becke, A. D. (1993). *J. Chem. Phys.* **98**, 5648–5652.

[bb4] Brandenburg, K. & Putz, H. (2012). *DIAMOND*, Crystal Impact GbR, Bonn, Germany.

[bb5] Bruker (2016). *APEX3*, *SAINT* and *SADABS*. Bruker AXS, Inc., Madison, Wisconsin, USA.

[bb6] Ellouz, M., Elmsellem, H., Sebbar, N. K., Steli, H., Al Mamari, K., Nadeem, A., Ouzidan, Y., Essassi, E. M., Abdel-Rahaman, I. & Hristov, P. (2016*b*). *J. Mater. Environ. Sci.* **7**, 2482–2497.

[bb7] Ellouz, M., Sebbar, N. K., Boulhaoua, M., Essassi, E. M. & Mague, J. T. (2017*a*). *IUCr Data* **2**, x170646.

[bb8] Ellouz, M., Sebbar, N. K., Elmsellem, H., Steli, H., Fichtali, I., Mohamed, A. M. M., Mamari, K. A., Essassi, E. M. & Abdel-Rahaman, I. (2016*a*). *J. Mater. Environ. Sci.* **7**, 2806–2819.

[bb9] Ellouz, M., Sebbar, N. K., Essassi, E. M., Ouzidan, Y. & Mague, J. T. (2015*a*). *Acta Cryst.* E**71**, o1022–o1023. 10.1107/S2056989015022987 PMC4719958 26870477

[bb10] Ellouz, M., Sebbar, N. K., Essassi, E. M., Ouzidan, Y., Mague, J. T. & Zouihri, H. (2016*c*). *IUCrData*, **1**, x160764.

[bb11] Ellouz, M., Sebbar, N. K., Essassi, E. M., Saadi, M. & El Ammari, L. (2015*b*). *Acta Cryst.* E**71**, o862–o863. 10.1107/S2056989015019295 PMC4645059 26594566

[bb12] Ellouz, M., Sebbar, N. K., Ouzidan, Y., Essassi, E. M. & Mague, J. T. (2017*b*). *IUCrData*, **2**, x170097.

[bb13] Ellouz, M., Sebbar, N. K., Ouzidan, Y., Kaur, M., Essassi, E. M. & Jasinski, J. P. (2017*c*). *IUCrData*, **2**, x170870.

[bb14] Frisch, M. J., Trucks, G. W., Schlegel, H. B., Scuseria, G. E., Robb, M. A., Cheeseman, J. R., *et al.* (2009). *GAUSSIAN09*. Gaussian Inc., Wallingford, CT, USA.

[bb15] Fujimura, K., Ota, A. & Kawashima, Y. (1996). *Chem. Pharm. Bull.* **44**, 542–546. 10.1248/cpb.44.542 8882451

[bb16] Groom, C. R., Bruno, I. J., Lightfoot, M. P. & Ward, S. C. (2016). *Acta Cryst.* B**72**, 171–179. 10.1107/S2052520616003954 PMC4822653 27048719

[bb17] Gupta, R. R. & Kumar, R. (1986). *J. Fluor. Chem.* **31**, 19–24.

[bb18] Hathwar, V. R., Sist, M., Jørgensen, M. R. V., Mamakhel, A. H., Wang, X., Hoffmann, C. M., Sugimoto, K., Overgaard, J. & Iversen, B. B. (2015). *IUCrJ*, **2**, 563–574. 10.1107/S2052252515012130 PMC4547824 26306198

[bb19] Hirshfeld, H. L. (1977). *Theor. Chim. Acta*, **44**, 129–138.

[bb20] Hni, B., Sebbar, N. K., Hökelek, T., Ouzidan, Y., Moussaif, A., Mague, J. T. & Essassi, E. M. (2019). *Acta Cryst.* E**75**, 372–377. 10.1107/S2056989019002354 PMC6399692 30867952

[bb21] Jayatilaka, D., Grimwood, D. J., Lee, A., Lemay, A., Russel, A. J., Taylor, C., Wolff, S. K., Cassam-Chenai, P. & Whitton, A. (2005). *TONTO - A System for Computational Chemistry.* Available at: http://hirshfeldsurface.net/

[bb22] Krause, L., Herbst-Irmer, R., Sheldrick, G. M. & Stalke, D. (2015). *J. Appl. Cryst.* **48**, 3–10. 10.1107/S1600576714022985 PMC4453166 26089746

[bb23] Malagu, K., Boustie, J., David, M., Sauleau, J., Amoros, M., Girre, R. L. & Sauleau, A. (1998). *Pharm. Pharmacol. Commun.* **4**, 57–60.

[bb24] McKinnon, J. J., Jayatilaka, D. & Spackman, M. A. (2007). *Chem. Commun.* pp. 3814. 10.1039/b704980c 18217656

[bb25] Merola, J. S. (2013). Private Communication (refcode 977080). CCDC, Cambridge, England.

[bb26] Rathore, B. S. & Kumar, M. (2006). *Bioorg. Med. Chem.* **14**, 5678–5682. 10.1016/j.bmc.2006.04.009 16650998

[bb27] Sabatini, S., Kaatz, G. W., Rossolini, G. M., Brandini, D. & Fravolini, A. (2008). *J. Med. Chem.* **51**, 4321–4330. 10.1021/jm701623q 18578473

[bb28] Sebbar, N. K., El Fal, M., Essassi, E. M., Saadi, M. & El Ammari, L. (2014*b*). *Acta Cryst.* E**70**, o686. 10.1107/S160053681401054X PMC4051051 24940264

[bb29] Sebbar, N. K., Ellouz, M., Boulhaoua, M., Ouzidan, Y., Essassi, E. M. & Mague, J. T. (2016*d*). *IUCrData*, **1**, x161823.

[bb30] Sebbar, N. K., Ellouz, M., Essassi, E. M., Saadi, M. & El Ammari, L. (2015). *Acta Cryst.* E**71**, o423–o424. 10.1107/S2056989015009755 PMC4459330 26090204

[bb31] Sebbar, N. K., Ellouz, M., Essassi, E. M., Saadi, M. & El Ammari, L. (2016*b*). *IUCrData*, **1**, x161012.

[bb32] Sebbar, N. K., Ellouz, M., Lahmidi, S., Hlimi, F., Essassi, E. & Mague, J. T. (2017*a*). *IUCrData*, **2**, x170695.

[bb33] Sebbar, N. K., Ellouz, M., Mague, J. T., Ouzidan, Y., Essassi, E. M. & Zouihri, H. (2016*c*). *IUCrData*, **1**, x160863.

[bb34] Sebbar, N. K., Ellouz, M., Ouzidan, Y., Kaur, M., Essassi, E. M. & Jasinski, J. P. (2017*b*). *IUCr Data* **2**, x170889.

[bb35] Sebbar, N. K., Mekhzoum, M. E. M., Essassi, E. M., Zerzouf, A., Talbaoui, A., Bakri, Y., Saadi, M. & Ammari, L. E. (2016*a*). *Res. Chem. Intermed.* **42**, 6845–6862.

[bb36] Sebbar, N. K., Zerzouf, A., Essassi, E. M., Saadi, M. & El Ammari, L. (2014*a*). *Acta Cryst.* E**70**, o614. 10.1107/S1600536814009179 PMC4011274 24860405

[bb37] Sheldrick, G. M. (2008). *Acta Cryst.* A**64**, 112–122. 10.1107/S0108767307043930 18156677

[bb38] Sheldrick, G. M. (2015*a*). *Acta Cryst.* A**71**, 3–8.

[bb39] Sheldrick, G. M. (2015*b*). *Acta Cryst.* C**71**, 3–8.

[bb40] Spackman, M. A. & Jayatilaka, D. (2009). *CrystEngComm*, **11**, 19–32.

[bb41] Spackman, M. A., McKinnon, J. J. & Jayatilaka, D. (2008). *Cryst. Eng. Comm*, **10**, 377–388.

[bb42] Takemoto, I., Yamasaki, K. & Kaminaka, H. (1994). *Biosci. Biotechnol. Biochem.* **58**, 788–789.

[bb43] Tawada, H., Sugiyama, Y., Ikeda, H., Yamamoto, Y. & Meguro, K. (1990). *Chem. Pharm. Bull.* **38**, 1238–1245. 10.1248/cpb.38.1238 2118427

[bb44] Turner, M. J., McKinnon, J. J., Wolff, S. K., Grimwood, D. J., Spackman, P. R., Jayatilaka, D. & Spackman, M. A. (2017). *CrystalExplorer17*. The University of Western Australia.

[bb45] Venkatesan, P., Thamotharan, S., Ilangovan, A., Liang, H. & Sundius, T. (2016). *Spectrochim. Acta Part A*, **153**, 625–636. 10.1016/j.saa.2015.09.002 26452098

[bb46] Vidal, A., Madelmont, J. C. & Mounetou, E. A. (2006). *Synthesis*, pp. 591–593.

[bb47] Wammack, R., Remzi, M., Seitz, C., Djavan, B. & Marberger, M. (2002). *Eur. Urol.* **41**, 596–601. 10.1016/s0302-2838(02)00174-4 12074775

[bb48] Warren, B. K. & Knaus, E. E. (1987). *Eur. J. Med. Chem.* **22**, 411–415.

[bb49] Zerzouf, A., Salem, M., Essassi, E. M. & Pierrot, M. (2001). *Acta Cryst.* E**57**, o498–o499.

[bb50] Zia-ur-Rehman, M., Choudary, J. A., Elsegood, M. R. J., Siddiqui, H. L. & Khan, K. M. (2009). *Eur. J. Med. Chem.* **44**, 1311–1316. 10.1016/j.ejmech.2008.08.002 18804313

